# Mental Healthcare Providers Understanding and Experiences of Palliative Care: A Qualitative Analysis

**DOI:** 10.1177/08258597221134865

**Published:** 2022-10-20

**Authors:** Tanya Park, Lydia Mutoni, Ramya Sridhar, Kathy Hegadoren, Bernadette Workun

**Affiliations:** 1 Faculty of Nursing, University of Alberta, Edmonton, AB, Canada; 2 3146 Alberta Health Services, Edmonton, AB, Canada

**Keywords:** mental health, palliative care, chronic persistent mental illness, nursing, qualitative research

## Abstract

**Objective:** To understand the experiences and perceptions of mental health providers about palliative care. **Background:** Little attention is paid to the experience of people with chronic persistent mental illness (CPMI) and life-threatening diseases and how their dying experience might differ from those without a CPMI. **Methods:** Interpretive description informed the project. Sixteen mental health care providers were interviewed using a semi-structured interview template. The interviews were recorded, transcribed, and analyzed using a reflexive, inductive-deductive thematic approach, guided by Braun & Clarke's framework for thematic analysis. **Results:** Four themes were identified from the data: intersectionality, limited collaboration, misconceptions about palliative care, and relationships. Mental health providers identified gaps in their knowledge of palliative care practices along with their knowledge of death and dying.

## Introduction

Little attention is paid to the experience of people with chronic persistent mental illness (CPMI) and life-threatening diseases and how their healthcare experience might differ from those without a CPMI. In Canada, one in five people experiences a mental illness in their life, despite age, education, and culture.^1^ Palliative care (PC) providers shared their challenges in meeting the palliative and mental health care needs of those with a CPMI.^2^ They expressed concerns related to a lack of resources and knowledge. Although their experiences indicated that this population group may represent a smaller proportion of patients in any given PC program, they can consume disproportionate resources.^2,3^ Similarly, in Ireland and Denmark studies found increasing rates of people with CPMI in PC services, with staff identifying knowledge deficits about mental illness and the associated treatments.^4,5,6^ A Canadian study found that people with CPMI are more likely to die in a nursing home with inadequate pain relief.^3^ This paper presents the findings of the second phase of a multiphase project to understand experiences and perceptions of providing care to people with a CPMI and life-threatening disease. This phase focused on understanding the perspectives and experiences of MHCPs.

Mental health and mental illness exist on a continuum. Mental health is understood as a state of well-being where “Every individual realizes his or her own potential, can cope with the normal stresses of life, can work productively and productively, and is able to contribute to … [one's] community”.^49^ Mental illnesses are disorders that affect a person's mood and affect, thinking, and behavior.^1^ CPMI refers to a smaller group of mental disorders, including schizophrenia, bipolar, and chronic major depressive disorder. These disorders have persistent symptoms that impede health and well-being, requiring ongoing support. Accessing mental health services regularly, developing and maintaining a therapeutic relationship with a consistent MHCP, increasing the risk of more frequent exacerbations of symptoms and hospitalizations is challenging, and living with a CPMI often requires a lifetime commitment to treatment.^7^ Treatments that are successful in minimizing psychotic or other chronic debilitating symptoms are also known to cause intrusive side effects such as extrapyramidal effects or metabolic problems.^5^ People with CPMI have a higher risk of developing life-limiting comorbidities such as cancer, cardiovascular disease (CVD), metabolic syndrome, diabetes, obesity, and smoking-related lung diseases.^8,9,10^ A person with schizophrenia is two to three times more likely to die from CVD and stroke, and they often die 25 years prematurely compared to the general population.^11,12,13^ For people with bipolar disorder and major depressive disorder, there is increased mortality linked to CVD, diabetes, and chronic obstructive pulmonary disease, with a reported premature loss of life of up to 8-9 years for people with bipolar disorder.^14,15^

Quality of life when living with a life-threatening disease is the focus of palliative care. The World Health Organization provides this comprehensive explanation: “Palliative care is an approach that improves the quality of life of people and their families who face problems associated with life-threatening diseases. Prevents and relieves suffering through early identification, correct assessment, and treatment of pain and other problems, whether physical, psychosocial, or spiritual”.^16^ There is no time limit to palliative care; life-limiting illnesses can be short; a few weeks or months and chronic where a person lives with a life-threatening illness for years. The WHO has estimated that ‘globally only 12% of patients who need palliative care receive it’.^16^ Canadian estimates are slightly higher with evidence suggesting that 15% of Canadians have access to quality palliative care.^17^ The aim of this study was to develop knowledge of the experience and perception of mental healthcare providers about palliative care for people living with a CPMI like schizophrenia. The literature confirmed that people with a CPMI do not frequently access palliative care services due to challenges often associated with their mental illness.^8,18,19^

## Methods

### Design

The Interpretive Description (ID) guided the design of the project. Thorne developed ID to satisfy the unique requirements of nursing science with the primary focus of understanding the experiences of the participants and ensuring that the findings are relevant to nursing practice.^20,21^ Ethical approval for this study was obtained from the Human Research Ethics Board of the University of XXXX. Informed consent was obtained verbally or in writing before the interview and participants were free to withdraw at any time before the start of data analysis. No incentives were offered for participation.

### Setting, Participants, and Data Collection

Participants were recruited from a variety of services in a large metropolitan city that provides all levels of mental health care. Health care settings included acute inpatient units, emergency departments, community agencies, and private practice. Recruitment was conducted from June to August 2020, using targeted purposeful and snowball recruitment strategies. The inclusion criteria for the participants were: (a) at least 18 years of age; (b) an MHCP; (c) experience caring for people diagnosed with a CPMI, and (d) English speaking.

*Sixteen* MHCP participants were interviewed. All semi-structured interviews were conducted online via a video call interface (Zoom) to ensure that all public health restrictions of the COVID-19 pandemic were met. The interview guide was derived from the phase 1 project with minor alterations to incorporate the focus on mental HCPs. The questions included *What has been your experience providing mental health care to someone with a CPMI? Please share a story about this? What barriers and facilitators have you encountered or are you aware of in providing mental health care to people with CPMI and advanced illness? How are palliative care services provided here?* The interviews lasted approximately one hour each. The interviews were recorded and transcribed with all identification information removed and replaced with numerical identification. The interviews were conducted by xx and xx under the supervision of xx. All interviews were conducted in English, this was the first language of all participants. After 16 interviews were completed the team agreed that data saturation had occurred and recruitment stopped, as the participant's experiences that were being shared were similar and no new information was being shared.

## Data Analysis

Data analysis procedures followed ID guidelines and included an inductive approach, data immersion, and thematic analysis. The inductive approach to analysis focused on generating understanding from the collected data.^21^ The transcripts were read multiple times by the research team. Inductive analysis was supported by frequent check-ins, field notes, and memos written before and during data collection, transcription, and the analysis process. These strategies ensured trustworthiness and rigor during the analysis. The thematic analysis framework by Braun and Clarke was used with a focus on latent themes and included the following steps: data immersion, code creation, exploration of themes, revision, identification and clarification, and report completion.^22^

## Results

*Sixteen* MHCP participants were interviewed, including 12 registered nurses, 2 registered psychiatric nurses, 1 social worker, and 1 psychologist. All participants were working in mental health care in a large urban city. They were working in private practice, acute, emergency and community care. The participants had a variety of health care experiences ranging from 5 years to 25 years. Four themes were developed from the interview data: *intersectionality, limited collaboration, misconceptions about palliative care, and relationships*.

### Intersectionality

Participants described complex situations, intersections between the person with a CPMI, their particular challenges, and the social determinants of health (SDOH). Social determinants of health are the social and economic aspects of a person's life that intersect, affecting their health in a positive or negative way.^23,24^ Intersectionality, originally introduced by Crenshaw, was used to interpret the unique problems of race and gender that exist for black women.^25^ Intersectionality has evolved to describe how variables converge (intersect) to place people at an increased disadvantage within the healthcare system. For example, participants described circumstances of stigma and discrimination that led to unfair or inadequate treatment. One participant told us that *prejudice and stigma are right within our health care, not just out in the public. There are certain health care providers who are afraid of people with mental illness, do not want to work with people with mental illness, and will avoid having to interact with clients with mental illness* (P12). Another participant described their struggle of working within a stigmatizing system; *It is kind of one of those things that I would say is systemic. The assumptions that people make about people who struggle with a mental illness are very wide and very varied* *…* *there is almost sometimes a prejudgment. If a person shows up and is not well dressed or clean, there is this assumption in some way that they will be aggressive, criminals, liars, drug addicts* *…* *just because you look unkempt, [but] you can very well have an illness that has nothing to do with taking drugs* (P8). While another participant shared that *I think the stigma* *…* *it is treated like a revolving door. Like, fix them, get them out, and they can come back later* (P7).

Participants also shared their experiences on how labeling people affect their care. One participant described *that many people get lost once they are considered non-compliant [where] you read their discharge summaries that indicated* ‘They were a difficult patient to comply with treatment, we highly suspect this patient will not be compliant once out of the hospital’ … it's *the anticipation that … Oh, they are not going to be compliant, there is nothing we can do about it, that's just the way they are* (P4). Another participant shared that *many Aboriginal patients talked about how they were discriminated against in-hospital or by different services. Whether it be the doctor not assessing them or just being turned away or not actually hearing them out … *(P3).

Participants noted 12 issues related to SDOH including; income and income distribution, education, unemployment and job security, early childhood development, food insecurity, housing, social exclusions, social safety network, health services, aboriginal status, gender and disability. Participants described that most people were impacted by multiple SDOHs at the same time, intersecting and compounding the inequalities they faced. One participant described this as *I feel like we work really closely with social workers due to the transient population that we see. A lot of their concerns are not having a safe place to stay or food, just like your basic needs. And so with anyone, if you did not have your basic support set up, it is hard to take care of your mental health, so a lot of what we do is to ensure that they have income, housing and some sort of support network* (P2). Another participant told us that *I mean if someone has nowhere safe to live and is not eating … I mean I can do all the therapy I want, but they are hungry and tired and lonely and scared because they are not safe* (P14).

The intersection of education and health care was clear; *some of my clients have a great university education. I have two professors on my client list, but others have very little education, like grade two if they are lucky. And so, can they read? No, but let's give them more handouts and say* ‘well, here are your resources’ *[and] well that's not valuable … *(P8). The intersection between housing and health care was also clear, *many patients are homeless, are not capable of taking their medications daily, do not have a consistent lifestyle, lose them or [they are prescribed] to them so they have to pick them up daily and then they don't go, or they decide they don't care., and then* [they] *have many traumas in their lives, and basically they are at risk for [everything]* (P7).

### Limited Collaboration

Limited collaboration describes how the quality of interactions between health care professionals occurs. Participants noted that limited collaboration among health care providers in mental health and other fields would negatively impact the mental health population they serve. For some participants, they would become part of complex communication situations where there was a lack of communication among professionals and care coordination became fragmented. This caused moral distress for the HCP and poor patient outcomes. A participant described … they *had several difficulties to the point of needing intubation and ICU care and after the fifth time, [ICU staff] said ‘*we will not take him back, don't bother calling us if he is coding, don't bother calling us cause we are not coming’ (P8). The participant found this interaction with the ICU distressing *[saying] that no, we are no longer* [able] *to call the experts because the client cannot comply with our treatment, therefore we will not see him* (P8). While another participant noted that *we have one patient whom I wish would talk to us about dying, who is in end-stage liver failure and never gets the pain control she really needs* (P7). The participant told us that *the doctor says that they have talked to her about palliative care, but I don't think they have done a good job explaining what that means … It's such a good case where palliative care could be so helpful, but is not implemented in a way that the patient can access it … I just don't feel like her quality of life is optimized* (P7).

Another participant shared; *with any medical condition, it changes someone's life, changes someone's body, changes their perception of themselves and how they are with others, so I think it goes hand in hand. I think we don't walk around without our heads, that's how I go right, our head is attached to our body. So why is mental health separated from medical conditions* (P13). Separation of mental and medical healthcare was described as *our clientele is certainly prone to some of the diseases that others may not be, so cardiovascular stuff, so metabolic monitoring is important, but yet, it is interesting, there is really no allowance to be doing that kind of care and monitoring* (P8). Feeling constrained was also described … It is *really counterintuitive, you think a mental health clinic may have the latitude to do those types of thing, and at the moment that is just not the case* (P8).

### Misconceptions About Palliative Care

The participants shared that they personally did not have a good understanding of palliative care. An RN working as a mental health therapist shared how she had tried to *get a [consultation for] palliative care,* but the psychiatrist had responded with … *Well, he is not dying … *(P8). A participant shared how they rarely thought about the benefits of palliative care for their mental health patients … *that is not something we are really oriented or educated about in the area where I work … that would not be the first thing that would come to my mind if I were dealing with someone who would benefit from palliative services … that is not something we have ever discussed* (P4). How people access palliative care was also not easily available … *actually, yeah, I don't know the answer to that and probably should* (P7). Some participants acknowledged that despite years of experience in mental health, palliative care is not frequently discussed and admitted that it should be considered more extensively.

Normalizing palliative care conversations was difficult; *no one wants to have that conversation with someone so young* (P7). HCPs avoided these conversations while recognizing the need for it, *but I don't think that should stop us from trying, I think that we have to give people at least an open invitation to speak about these things that are maybe not comfortable, and particularly in mental health because we all know that if we have emotions and thoughts and don't deal with them, they tend to build up* (P8) and *as clinicians, we owe it to ourselves to be on top of … our own feelings, and our own biases, and our own thoughts on death and dying so that we can be open and give latitude to clients* (P7).

### Relationships

Social support networks are key elements in terms of quality of life. A participant noted that *if the individual cannot advocate for himself or have an advocate for him, like a family member who is, you know, advocating and getting the ball rolling, and getting the services … I think those individuals are at high risk of not getting the support that they need* (P12). The participants emphasized the positive difference that strong relationships can make … She was married, had a husband, had adult children, and although she had depression, I mean she had a fairly productive life up to her cancer diagnosis, and so she did have support. She had her family, she was receiving treatment … she died within a year, but she was one who had support (P12). Another participant noted … If individuals don'*t have the social support system, then clinical support [s] can make a big difference and vice versa* (P3).

With limited social support networks, developing and maintaining a strong sense of self can be challenging. One participant told us that *some of my clients are quite isolated. So they might have a [reservoir] of internal reserves in which to lean, but … some are isolated socially, so they don't have as many [supports]* (P16). A major consequence of this is a deteriorating sense of self, which also directly impacts the motivation to be health literate and actively participate in one's own care. One participant described … I *think someone who has a chronic and debilitating mental illness loses* *…* *they just are not active advocates for themselves. They either accept it or they learn that somehow they don't matter as much as anybody else* (P12). Another participant acknowledged that *self-esteem is really affected by the way society has reacted to them and the fact that they have been marginalized* *…* *they are not seen as a contributing member of society by many sectors* (P12).

## Discussion

The four themes that developed from the data were *intersectionality, limited collaboration, misconceptions of palliative care, and relationships*.

Social exclusion and biases are examples of SDOH that intersect to influence the quality of care. HCPs working in mental health care are not immune to carrying these social biases. Participants shared uncomfortable experiences in which they were reluctant to consult patients with mental health issues, highlighting the effect of stigma, discrimination, and knowledge deficits. These biases, whether conscious or unconscious, negatively influence the care that clients receive and delay transitions/referrals to specialist services.^9^ A recent systematic review of stigma and discrimination found that nurses and patients perceive similar barriers to care, leading to compromised care and relationships for people with mental illness.^26^ Tyerman et al reported individual, institutional, and social stigma and although our project did not categorize the stigma experienced or witnessed examples of stigma were shared throughout the interviews.^26^ Participants reported individual and institutional stigma, including circumstances of witnessing stigma and discriminating views that led to refusing to treat or providing inadequate treatment to people with mental illness.

Participants in this study shared with us how they experienced barriers to understanding and including palliative care in their practices. Participants reflected on their experiences working with people with mental illness isolated from receiving other care services. Collaborating and consulting with healthcare colleagues has been shown to work, providing much-needed background information to best manage patient needs.^18^ Even when services such as PC could be beneficial, participants described workplace hierarchies and power relationships within the healthcare system that prevented them from advocating and taking action. Hierarchies within the healthcare team and across the healthcare system are two consistent threads in the literature. Hierarchies have been shown to lead to potential harm to patients, particularly when there is poor communication and information sharing.^50^ Interprofessional collaboration has been a long-standing challenge for healthcare teams. In 1999, Peck & Norman reported on the experiences of an adult community mental health service and the challenges of interprofessional collaboration.^27^ They found that socialization and professional training influenced collaboration within the healthcare team. Despite awareness of the importance of interprofessional collaboration, we still have not rectified this issue and negative patient outcomes continue.^28–34^ The stories of the participants demonstrate how fractured healthcare systems can lead to limited collaboration within the health team and across the health system.

HCPs who work in mental health care will benefit from educational interventions to improve their PC knowledge. Misinterpretations were due to varying levels of PC knowledge and the need to normalize palliative care conversations. Due to the prevalence of comorbidities, it is essential for HCPs working in mental health to have a working knowledge of PC and increase competence in this area.^3,8,18,35,36^ Frequent assumptions about accessing palliative care included people who needed to be near death, associating it primarily with cancer diagnoses, and questioning its relevance for people with mental illness.^35,36^ Increasingly PC approaches in psychiatry are being discussed in the literature. ^37–39^ Highlighting the potential to improve the quality of life of people with CPMI by incorporating PC frameworks and standards such as care conversation goals.^38,39^ Participants discussed how HCPs participated in or did not clarify negative perceptions about PC, even when patients associated PC with giving up and loss of hope. Palliative care was not actively discussed for people with mental illness as a means to improve quality of life, increase symptoms and pain management, resolve patient-family conflicts, and prioritize comfort-focused measures.^40^

Relationships are fundamental to mental health nursing and mental health. Since Peplau wrote about the need to connect with the self and the other, we have highlighted the importance of relationships in mental healthcare.^41–46^ However, it is interesting that patients/clients and HCPs continue to struggle to develop supportive therapeutic relationships. In addition, interprofessional relationships are an ongoing source of discussion.^47^

Price et al reported that HCPs had difficulty communicating with patients, their families, and interprofessional teams, leading to improper decision-making and planning for palliative care.^48^ Price et al noted that HCPs needed educational guidance for end-of-life discussions that include the patient's and their family's wishes.^48^ Improving all relationships will lead to better patient outcomes.

Limitations for this project included adaptations that were made to accommodate the COVID-19 pandemic and public health restrictions. In-person interviews were replaced with video/audio communications, depending on the preferences of the participants. Recruitment was limited to electronic communication. Notably, three participants agreed to be interviewed and then withdrew citing work stress due to the pandemic. Despite this, we managed to recruit a variety of participants who had experience in various settings, including inpatient psychiatry, emergency, intensive care psychiatric, private practice, rehabilitation, geriatrics, and community, allowing for a breadth of unique experiences.

In conclusion, it is clear from this project that there is an urgent need to share information and improve the knowledge of health care providers related to all aspects of PC. Educational activities in the areas of intersectionality, unconscious bias training, and palliative care would improve understanding of palliative care with diverse patient populations and lead to improved patient outcomes. Interprofessional collaboration should be an ongoing focus in the development of teams. Valuing interprofessional knowledge is the first step in improving collaboration. Regular communication among HCPs needs to improve. The first phase of this project was to understand the perspectives of palliative care providers. This second phase looked at the perspectives of mental health care providers. The third phase will focus on the experiences of patients and their families. The broad aim is to co-develop and test a model of care to guide palliative care for patients with CPMI and advanced disease.

Implications for healthcare practice include highlighting the challenges experienced by HCP can encourage reflexive practice. The findings identified education gaps in the areas of palliative care, relational practice, health literacy, intersectionality, unconscious bias, death and dying. There are opportunities to improve current nursing education by incorporating educational opportunities, such as implicit bias training, to learn how to identify and navigate our unconscious biases.
